# Network-based SNP meta-analysis identifies joint and disjoint genetic features across common human diseases

**DOI:** 10.1186/1471-2164-13-490

**Published:** 2012-09-18

**Authors:** Matthias Arnold, Mara L Hartsperger, Hansjörg Baurecht, Elke Rodríguez, Benedikt Wachinger, Andre Franke, Michael Kabesch, Juliane Winkelmann, Arne Pfeufer, Marcel Romanos, Thomas Illig, Hans-Werner Mewes, Volker Stümpflen, Stephan Weidinger

**Affiliations:** 1 Institute of Bioinformatics and Systems Biology, Helmholtz Zentrum München, German Research Center for Environmental Health, 85764, Neuherberg, Germany; 2 Department of Dermatology and Allergy Biederstein, Technische Universität München, 80802, Munich, Germany; 3 TUM Graduate School of Information Science in Health (GSISH), Technische Universität München, 85748, Garching, Germany; 4 Institute for Clinical Molecular Biology, University of Kiel, 24105, Kiel, Germany; 5 Clinic for Pneumology and Neonatology, Hannover Medical School, 30625, Hannover, Germany; 6 Institute for Human Genetics, Technische Universität, München, 81675, Munich, Germany; 7 Department of Neurology, Technische Universität München, 81675, Munich, Germany; 8 Institute of Human Genetics, Helmholtz Zentrum München, German Research Center for Environmental Health, 85764, Neuherberg, Germany; 9 Institute of Genetic Medicine, European Academy Bozen/Bolzano (EURAC), 39100 Bolzano, Italy – Affiliated Institute of the University Lübeck, 23562, Lübeck, Germany; 10 Department of Child and Adolescent Psychiatry, University Clinic of Munich, 80336, Munich, Germany; 11 Institute of Epidemiology, Helmholtz Zentrum München, German Research Center for Environmental Health, 85764, Neuherberg, Germany; 12 Chair of Genome Oriented Bioinformatics, Center of Life and Food Science, Freising-Weihenstephan, Technische Universität München, 80333, Munich, Germany

**Keywords:** Genome-wide association study, Genetic overlap, Shared variant network, Disease comorbidity

## Abstract

**Background:**

Genome-wide association studies (GWAS) have provided a large set of genetic loci influencing the risk for many common diseases. Association studies typically analyze one specific trait in single populations in an isolated fashion without taking into account the potential phenotypic and genetic correlation between traits. However, GWA data can be efficiently used to identify overlapping loci with analogous or contrasting effects on different diseases.

**Results:**

Here, we describe a new approach to systematically prioritize and interpret available GWA data. We focus on the analysis of joint and disjoint genetic determinants across diseases. Using network analysis, we show that variant-based approaches are superior to locus-based analyses. In addition, we provide a prioritization of disease loci based on network properties and discuss the roles of hub loci across several diseases. We demonstrate that, in general, agonistic associations appear to reflect current disease classifications, and present the potential use of effect sizes in refining and revising these agonistic signals. We further identify potential branching points in disease etiologies based on antagonistic variants and describe plausible small-scale models of the underlying molecular switches.

**Conclusions:**

The observation that a surprisingly high fraction (>15%) of the SNPs considered in our study are associated both agonistically and antagonistically with related as well as unrelated disorders indicates that the molecular mechanisms influencing causes and progress of human diseases are in part interrelated. Genetic overlaps between two diseases also suggest the importance of the affected entities in the specific pathogenic pathways and should be investigated further.

## Background

In the past years enormous progress has been made in the identification of complex trait susceptibility loci. The application of genome-wide association studies (GWAS) created a still growing set of genetic markers associated with increased risk for a multitude of different diseases [1]. In contrast to few single loci exerting large effects on some phenotypes – mostly immune-related traits – the majority of traits was only associated with loci displaying small effects of odds ratios ranging from 1.1 to 1.5 [2]. Meta-analyses of several GWA studies further extended the set of known disease-related associations with even lower-effect variants. Despite the impressive progress in the field, for most traits only a small proportion of the total heritability is yet explained by known risk variants [3]. A notable exception is type 1 diabetes (T1D) where validated risk loci explain a large proportion of the total heritability [4]. In contrast, for most traits a considerably larger number of variants was reported to be associated, but typically these explain less than 50% of the total heritability [5].

Intriguingly, although published individual GWAS are usually carried out for one trait at a time, a significant overlap in the associations of several complex diseases becomes apparent [6]. Besides effects on a specific phenotype, loci and single SNPs thus may also exert pleiotropic effects by contributing to a variety of traits. While it is not surprising that susceptibility genes for closely related traits should be shared, multi-functionality of a gene in phenotype presentation, i.e. pleiotropy, *sensu stricto* refers to seemingly unrelated and distinct traits [7]. Loci or variants affecting several traits might have small effects on each specific trait, but may be of major biological interest while indicating shared or branching etiological mechanisms. In principle, the influence of such loci can be agonistic or antagonistic, i.e. involve concurrent similar or opposite effects of the same variant for different traits. So far, few studies attempted to study such loci in a systemic fashion and rather focused on shared risk variants in closely related traits like autoimmune diseases [8-10], heart diseases [11] or cancer [12].

In order to identify shared or branching pathways of related as well as diverse (i.e. medically and phenotypically distinct) diseases, we performed a systematic comparative analysis of genetic commonalities and differences across traditionally defined traits using the available repository of GWAS results. In the context of network medicine [13], we utilized an approach based on the diseasome concept [14] and investigated high-significance associations beyond conventional single-marker analysis in a hypothesis-free and comprehensive way. In former studies we found differing approaches of gene and locus assignment to association markers which partially led to controversial results (e.g. [15]). We therefore developed a more sophisticated locus assignment method and evaluate its reliability by utilizing the information contained directly in the reported markers. For this variant-based approach we manually curated a high-quality data set to construct a network extending the knowledge on genetic overlaps between diseases as provided by GWA studies.

## Results and discussion

**Figure 1:**
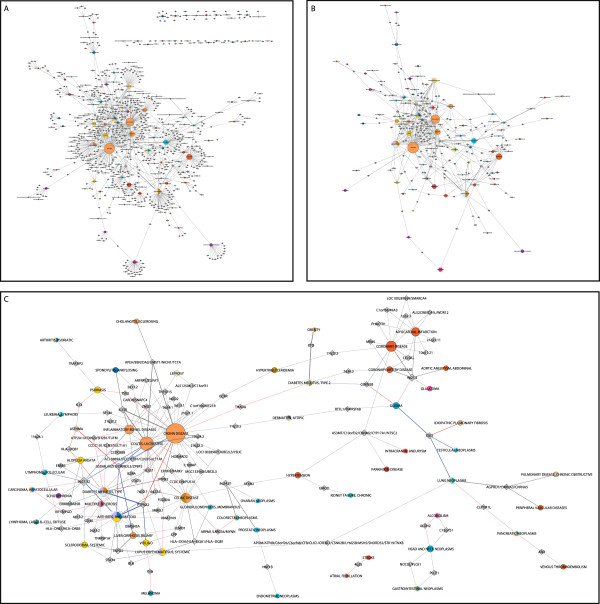
**Illustration of the different disease networks based on genome-wide association data.A**: The bipartite graph constructed from all association data. The two disjoint node sets are diseases (n = 111) and loci (n = 734; 508 gene loci and 226 intergenic loci), connected to each other by an edge if a variant (n = 1,120) within the respective locus is associated with the corresponding trait. **B**: The SLN (shared locus network) consisting of 84 traits and 157 loci, retrieved by removing isolated traits and loci that are associated with a single trait only. **C**: The SVN (shared variant network) that corresponds to a variant-based representation of the data. Here, a trait and a locus are linked if the locus contains a variant comprising associations with this and at least one other trait. The network consists of 175 SNPs located in 94 loci that are associated with 55 diseases (see also Additional file 2: Table S1). The colors of the disease nodes correspond to disease classes according to the MeSH ontology, multi-colored nodes indicate an association with different disease classes; loci are depicted as transparent, diamond-shaped nodes. The node size reflects the number of loci a disease is associated with. In C, the edge color reflects the allelic information: gray indicates agonistic variant(s), red corresponds to antagonistic variant(s), and blue mark both agonistic and antagonistic signals.

Considerable discrepancies across GWAS through differing genotyping platforms, varying sample sizes and diverging measures of statistical significance demand accurate data selection. Therefore, to sustain the genuine variant-linked information provided by GWAS, we combined several steps of data curation and filtering. To provide a comprehensive base for the analysis of potentially multi-functional loci and variants, respectively, we compiled two network representations of the information made available by GWA studies: the locus-based “shared locus network” (SLN, Figure 1B) and the variant-based “shared variant network” (SVN, Figure 1C). To be able to cluster diseases by their “genetic relatedness”, we additionally created a disease-centric projection of the SVN (Additional file 1: Figure S1).

We defined the associated loci over the variant-based linkage disequilibrium (LD) measure *r*^2^ and, accordingly, expected the SLN and the SVN (Figure 1B and C) to be of similar shape. However, when visually comparing the networks, significant differences in size and structure became apparent. Therefore, we performed further analyses to compare established property measures of the networks in detail to investigate potential reasons for this divergence.

### Shared locus vs. shared association analysis

**Figure 2:**
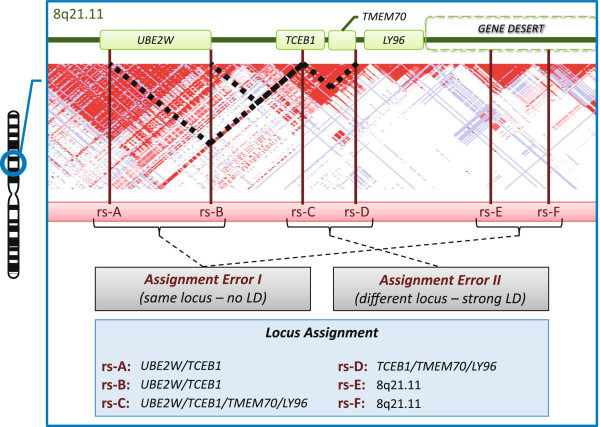
**LD based locus assignment and its error sources.** At the example of chromosome 8q21.11, LD-based locus assignment is given for 6 exemplary SNPs (blue box). LD information is given by a color scale displaying the LD-measure *r*^2^ with red depicting strong LD, blue low LD and white no LD. Example SNPs in LD are connected with black dashed lines. In the gray boxes, the two error sources of automated locus assignment are given. An assignment error I occurs if two variants not in LD, i.e. in two independent LD blocks, are located in the same gene, intergenic region or gene desert and thus are assigned the same locus. Here, this is the case for the variants rs-A/rs-B and rs-E/rs-F, respectively. The consequence of this type of error is a shared association on the locus level not mirrored on the variant level. An assignment error II is introduced if two variants are in LD but diverge in their assigned locus. Here, this is the case for rs-C and rs-D. Due to such abnormalities in the LD data the link between both variants is lost if only the locus level is considered.

Despite the size difference, the SLN shows greater network heterogeneity (*SLN* = 1.30, *SVN* = 1.17) and lower centralization (*SLN* = 0.175, *SVN* = 0.205) and density (*SLN* = 0.014, *SVN* = 0.021) values than the SVN. Furthermore, the intersection between the SVN and the SLN lacks not only 5% of the nodes but also 10% of the edges of the SVN. These numbers imply that the process of translating LD data into locus information is at least partly inconsistent. Analysis of the structure of the assigned LD blocks showed two error sources in shared locus analysis (illustrated in Figure 2). First, variants in two independent LD blocks are assigned the same locus but are not in LD (assignment error I). Thus, shared loci are found that are not reflected in the variant based data. Second, if two SNPs are in strong LD but the individual long range LD patterns of the SNPs diverge (e.g. the LD block of one SNP covers a greater area at the given *r*^2^-threshold), an assignment error II occurs. In this case the two SNPs are assigned to different loci (in the example above, this is due to the different sizes of their LD blocks which may contain distinct gene sets) and their LD connection is lost. These observations suggest that (*i*), the SLN contains loci which overlap between traits but the associated markers are not in strong LD, (*ii*), there are several traits which are connected to the SLN via a single, potentially misleading link (as not mirrored in the variant-based data), and, (*iii*), even a LD-based locus assignment is unable to identify all shared associations (*n* = 25 of unidentified loci, based on the assignment error II). Due to this limited sensitivity and specificity in detecting LD-based correlations between the reported markers on locus scale, we used the smaller but more accurate variant-based SVN for further analyses. Moreover, the risk allele data in the SVN allows for the inclusion of the direction of the effects, i.e. agonistic and antagonistic, on different traits. Diseases that share antagonistic or agonistic, respectively, associated variants are listed in Tables 1 and 2. Another advantage is that the SVN can be compared to other variant-based approaches assessing the genetic overlap between traits. Recently, a statistic to identify SNPs with effects across phenotypes (the cross-phenotype meta-analysis statistic, CPMA) was proposed by Cotsapas et al. [8]. It compares the distribution of association P-values of a SNP across seven GWAS on distinct autoimmune diseases to the exponential(1)-distribution representing the expected decay rate of association P-values. As in our approach we use pre-filtered associations, this method cannot readily be employed on our data. However, using the data provided by Cotsapas and colleagues on autoimmune loci in the SVN, we retrieved CPMA P-values on 30 SNPs (∼ 17%) corresponding to 28 loci (∼ 30%) in our data. The CPMA classified all SNPs as significantly effective across diseases (*P* < 0.05, see Additional file 3: Table S2). Thus, we were able to validate nearly one third of the loci contained in the SVN by an independent approach, which underlines the suitability of the network.

**Table 1:** Antagonistically linked traits

**Disease**	**Abbr.**	**Antagonistically Linked Traits (*Loci*)**	**#**
**CROHN DISEASE**	CD	AST (*17q12*); T1D (*1p13, 16p11*); VIT (*1p13*); T2D (*2p21*); RA (*1p13*); SLE (*1p13*); PS (*19p13*); MS (*17q21*)	8
**ASTHMA**	AST	CD (*17q12*); UC (*17q12*); BLC (*17q12*); RA (*17q12*); PS (*5q31*)	5
**GLIOMA**	GLI	COR (*9p21*); T2D (*9p21*); GLA (*9p21*); IPF (*5p15*) ; TN (*5p15*)	5
**ARTHRITIS, RHEUMATOID**	RA	AST (*17q12*); MS (*20q13*); CD (*1p13*)	3
**MULTIPLE SCLEROSIS**	MS	RA (*20q13*); HCC (*1p36*); CD (*17q21*)	3
**COLITIS, ULCERATIVE**	UC	AST (*17q12*); CeD (*1p36*); SLE (*1q23*)	3
**LUNG NEOPLASMS**	LN	PN (*5p15*); IPF (*5p15*); TN (*5p15*)	3
**TESTICULAR NEOPLASMS**	TN	GLI (*5p15*); LN (*5p15*)	2
**VITILIGO**	VIT	CD (*1p13*); MEL (*11q14*)	2
**DIABETES MELLITUS, TYPE 1**	T1D	CD (*1p13, 16p11*); IBD (*1p13*)	2
**DIABETES MELLITUS, TYPE 2**	T2D	GLI (*9p21*); CD (*2p21*)	2
**CELIAC DISEASE**	CeD	UC (*1p36*); CRC (*3q26*)	2
**IDIOPATHIC PULMONARY FIBROSIS**	IPF	GLI (*5p15*); LN (*5p15*)	2
**PSORIASIS**	PS	AST (*5q31*); CD (*19p13*)	2
**LUPUS ERYTHEMATOSUS, SYSTEMIC**	SLE	CD (*1p13*); UC (*1q23*)	2
**GLAUCOMA**	GLA	GLI (*9p21*)	1
**MELANOMA**	MEL	VIT (*11q14*)	1
**PANCREATIC NEOPLASMS**	PN	LN (*5p15*)	1
**ALCOHOLISM**	ALC	HNN (*12q24*)	1
**COLORECTAL NEOPLASMS**	CRC	CeD (*3q26*)	1
**INFLAMMATORY BOWEL DISEASES**	IBD	T1D (*16p11*)	1
**CARCINOMA, HEPATOCELLULAR**	HCC	MS (*1p36*)	1
**LIVER CIRRHOSIS, BILIARY**	BLC	AST (*17q12*)	1
**CORONARY DISEASE**	COR	GLI (*9p21*)	1
**HEAD AND NECK NEOPLASMS**	HNN	ALC (*12q24*)	1

Listed are diseases and the traits that share an antagonistic variant with the respective disorder. In the first column the considered disease is given. The second column specifies the abbreviation of the disorder in the first column. The third column contains the abbreviated diseases as defined in column two which have an antagonistic link to the disorder in column one, followed by the chromosomal location of the antagonistically associated variant(s) in parentheses. The last column lists the count of traits antagonistically linked to the disorder in column one. For a more detailed listing see Additional file 2: Table S1.

**Table 2:** Agonistically linked traits

**Disease**	**Abbr.**	**Agonistically Linked Traits (*Loci*)**	**#**
**CROHN DISEASE**	CD	UC (*1p31, 1q32, 3p21, 5p13, 9p24, 9q32, 9q34, 10q24, 21q21, 21q22*); IBD (*1p31, 9q32, 10q22, 16q12, 20q13, 22q12*); CeD (*2q12, 18p11, 22q11*); LEP (*9q32, 13q14*); MG (*22q12*); T1D (*1q32, 18p11*); SC (*3p21*); OVARIAN NEOPLASMS (*8q24*); AS (*1p31*); LL (*2q37*); HTG (*2p23*); AA (*11q13*); MS (*10p15*); AD (*11q13*)	14
**ARTHRITIS, RHEUMATOID**	RA	CeD (*1p36, 4q27, 6q23*); SLE (*2q32, 7q32, 8p23*); T1D (*2q33, 4p15, 21q22*); BLC (*7q32*); SS (*7q32*); FL (*6p21*); UC (*6q23*); LBL (*6p21*); SCHIZOPHRENIA (*6p21*); VIT (*21q22*)	10
**CELIAC DISEASE**	CeD	CD (*2q12, 18p11, 22q11*); RA (*1p36, 4q27, 6q23*); UC (*2p16, 6q23*); T1D (*18p11*); BLC (*7p14*); VIT (*3q28*); HYP (*12q24*); SLE (*6p21*); MG (*6p21*)	9
**COLITIS, ULCERATIVE**	UC	CD (*1p31, 1q32, 3p21, 5p13, 9p24, 9q32, 9q34, 10q24, 21q21, 21q22*); CeD (*2p16, 6q23*); AS (*1p31, 2q11*); IBD (*1p31, 21q22*); SC (*3p21*); PS (*1p31*); RA (*6q23*); T1D (*1q32*); SLE (*7q32*)	9
**CORONARY DISEASE**	COR	MCI (*1p13, 1p32, 1q41, 2q33, 6p24, 9p21, 10q11, 19p13, 21q22*); CAD (*1p13, 3q22, 9p21*); ICA (*10q24*); PD (*10q24*); AAA (*9p21*); HYP (*10q24*); HTG (*11q23*)	7
**LUPUS ERYTHEMATOSUS, SYSTEMIC**	SLE	RA (*2q32, 7q32, 8p23*); SS (*2q32, 7q32*); LN (*6p21*); BLC (*7q32*); MG (*6p21*); UC (*7q32*); CeD (*6p21*)	7
**DIABETES MELLITUS, TYPE 1**	T1D	RA (*2q33, 4p15, 21q22*); CD (*2q33, 4p15, 21q22*); CeD (*18p11*); AA (*12q13*); VIT (*21q22*); UC (*1q32*)	6
**HYPERTENSION**	HYP	ICA (*10q24*); PD (*10q24*); COR (*10q24*); KIDNEY FAILURE, CHRONIC (*16p12*); CeD (*12q24*)	5
**INFLAMMATORY BOWEL DISEASES**	IBD	CD (*1p31, 9q32, 10q22, 16q12, 20q13, 22q12*); LEP (*9q32*); UC (*1p31, 21q22*); MG (*22q12*); AS (*1p31*)	5
**LIVER CIRRHOSIS, BILIARY**	BLC	RA (*7q32*); SLE (*7q32*); SS (*7q32*); MS (*12p13*); CeD (*7p14*)	5
**CORONARY ARTERY DISEASE**	CAD	COR (*1p13, 3q22, 9p21*); MCI (*1p13, 9p21*); AAA (*9p21*); T2D (*2q36*)	4
**GLOMERULONEPHRITIS, MEMBRANOUS**	MG	IBD (*22q12*); CD (*22q12*); SLE (*6p21*); CeD (*6p21*)	4
**AORTIC ANEURYSM, ABDOMINAL**	AAA	COR (*9p21*); CAD (*9p21*); MCI (*9p21*)	3
**INTRACRANIAL ANEURYSM**	ICA	PD (*10q24*); HYP (*10q24*); COR (*10q24*)	3
**LUNG NEOPLASMS**	LN	SLE (*6p21*); COPD (*15q25*); PVD (*15q25*)	3
**LYMPHOMA, FOLLICULAR**	FL	LBL (*11q24*); RA (*6p21*); LL (*6p21*)	3
**MYOCARDIAL INFARCTION**	MCI	COR (*1p13, 1p32, 1q41, 2q33, 6p24, 9p21, 10q11, 19p13, 21q22*); CAD (*1p13, 9p21*); AAA (*9p21*)	3
**PARKINSON DISEASE**	PD	HYP (*10q24*); COR (*10q24*); ICA (*10q24*)	3
**SCLERODERMA, SYSTEMIC**	SS	SLE (*2q32, 7q32*); RA (*7q32*); BLC (*7q32*)	3
**SPONDYLITIS, ANKYLOSING**	AS	UC (*1p31, 2q11*); IBD (*1p31*); CD (*1p31*)	3
**VITILIGO**	VIT	CeD (*3q28*); RA (*21q22*); T1D (*21q22*)	3
**ALOPECIA AREATA**	AA	T1D (*12q13*); CD (*11q13*)	2
**CHOLANGITIS, SCLEROSING**	SC	UC (*3q21*); CD (*3q21*)	2
**DERMATITIS, ATOPIC**	AD	GLIOMA (*20q13*); CD (*11q13*)	2
**DIABETES MELLITUS, TYPE 2**	T2D	OBESITY (*16q12*); CAD (*2q36*)	2
**HEAD AND NECK NEOPLASMS**	HNN	GASTROINTESTINAL NEOPLASMS (*10q23*); ALCOHOLISM (*12q24*)	2
**HYPERTRIGLYCERIDEMIA**	HTG	CD (*2p23*); COR (*11q23*)	2
**LEPROSY**	LEP	CD (*9q32, 13q14*); IBD (*9q32*)	2
**LEUKEMIA, LYMPHOID**	LL	CD (*2q27*); FL (*11q24*)	2
**LYMPHOMA, LARGE B-CELL, DIFFUSE**	LBL	FL (*6q21*); RA (*6q21*)	2
**MULTIPLE SCLEROSIS**	MS	CD (*10p15*); BLC (*12p13*)	2
**PERIPHERAL VASCULAR DISEASES**	PVD	COPD (*15q25*); LN (*15q25*)	2
**PROSTATIC NEOPLASMS**	PRN	COLORECTAL NEOPLASMS (*8q24*); ENDOMETRIAL NEOPLASMS (*17q12*)	2
**PSORIASIS**	PS	UC (*1p31*); ARTHRITIS, PSORIATIC (*6q21*)	2
**PULMONARY DISEASE, CHRONIC OBSTRUCTIVE**	COPD	LN (*15q25*); PVD (*15q25*)	2

Listed are diseases that share agonistic associations with at least two traits. In the first column the considered disease is given. The second column specifies the abbreviation of the disorder in the first column. The third column contains the disease abbreviations (as defined in column two) of traits which have an agonistic link to the disorder in column one, followed by the chromosomal location of the agonistically associated variant(s) in parentheses. Here, the full MeSH term is given for traits for which no abbreviation was defined. The last column lists the count of traits agonistically linked to the disorder in column one. For the complete list of agonistically linked traits and more details see Additional file 2: Table S1.

### Topology of the SVN

Its degree distribution attributes the SVN a scale-free network, i.e. it approximates a power-law (*P*(*k*) ∼ *k*^−*γ*^; *γ* = 1.32; *R*^2^ = 0.69) (Additional file 4: Figure S2). Interestingly, also when considering the two node types separately, disease nodes (*γ* = 0.97; *R*^2^ = 0.71) as well as locus nodes (*γ* = 2.98; *R*^2^ = 0.93) show scale-free degree distributions (Additional file 4: Figure S2). The scale-free property classifies the network (and its two sets of node types, respectively) as structured, i.e. non-random [13]. It has to be considered that the limited size of the SVN leads to inaccuracies in distribution fitting and thus reduces the explanatory value of this observation. However, as clinically related diseases (i.e. diseases which present similar symptoms) should present a higher genetic overlap than unrelated disorders, this finding meets expectations.

The variant-based SVN also shows no artificial character with regards to its topology. Both locus and disease node sets comprise hubs, here defined as nodes with a degree >3, which form the central elements in the network. As in each GWAS multiple markers are associated with a single disease, one would expect hubs to be constituted mostly of disease nodes. In line with that, 74% of the hubs in our network are disease nodes. The remaining 26% are loci hubs (seven gene loci and three intergenic loci). Several of these loci have been previously identified as influencing susceptibility to multiple diseases like the HLA region on chromosome 6 [16], a cancer locus at chromosome 8q24 [12], and a coronary artery disease locus at chromosome 9p21 [11]. Further hub loci are *PTPN22*, a known player across several autoimmunity disorders [17], and *IL23R*, which has been shown to direct inflammatory processes [18]. In addition, we observed hubs which have not yet been described as predisposing to a whole group of diseases, such as *TNPO3* which appears to predispose to various autoimmune diseases like systemic lupus erythematosus, systemic scleroderma, and rheumatoid arthritis [19-21], or *TNFSF15*, which shows associations with several inflammatory diseases [22-25]. As expected, in the majority of cases the traits linked to one hub can be assigned to the same disease group and, further, diseases which are not obviously related to other disorders linked to the respective hub are mostly associated with antagonistic signals. For instance, in a four-gene locus at chromosome 17q12 (*GSDML*/*IKZF3*/*ORMDL3*/*ZPBP2*, see Additional file 2: Table S1), four autoimmune diseases are associated with the same risk allele that in turn has opposite effects on asthma [20,25-27]. Thus, our results indicate that loci associated with several diseases have an effect specific to a certain disease group rather than effects on unrelated diseases, and that, if there is an effect on an unrelated disease, it can often be distinguished by the direction of the effect.

### Disease clustering mirrors trait relatedness

To identify shared and branching mechanisms we split the SNP association data into agonistic and antagonistic variants. Since in most cases there is no solid and comprehensive basis of experimental data that would allow for a more sensitive classification, we suggest that the best available indication of distinct effects of a variant on two diseases is the signal itself being different. Therefore, we define a SNP to be agonistic if all disorders are associated with the same risk allele of the SNP. Conversely, we consider a SNP antagonistic if the associated risk alleles differ between diseases. Accordingly, in the analysis of genetic overlaps as a measure of trait similarity only agonistic variants were included.

**Figure 3:**
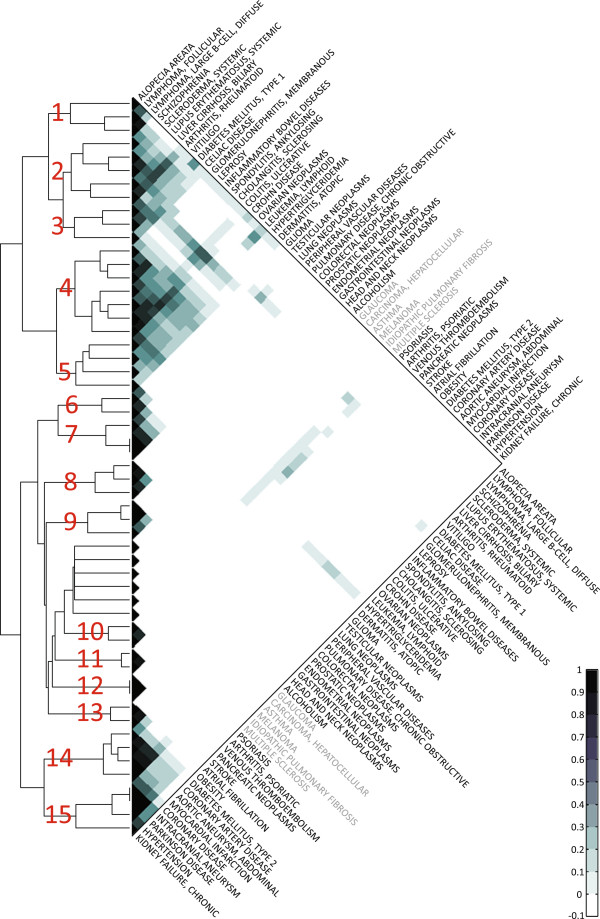
**Clustering of diseases with respect to genetic signals.** We applied complete-linkage hierarchical clustering to identify groups of traits which show homogeneous patterns of genetic overlap to other disorders. We calculated for each pair of diseases the Pearson correlation of the patterns of overlap to the other diseases. The correlation values are ranging from −1 (white) indicating complete negative correlation to +1 (black) reflecting a perfect positive correlation. As the minimal value of the correlation coefficient was > −0.1, we collapsed the range of negative correlation. In red numbers, the 15 disease clusters are denoted. The Euclidian distance threshold was chosen as the maximal distance at which the six diseases showing no or only weak correlation with any other disease (disease names in gray) remain non-clustered.

As similar diseases are more likely to share associations than diseases in distinct classes, we expected the SVN to be organized in a modular fashion. This was confirmed by the decrease of the degree distribution of the topological coefficient with the number of links per node (Additional file 4: Figure S2). To retrieve these modules, we applied a hierarchical clustering approach. The SVN contains two node types (loci and traits). As we wanted to directly assess variant-based disease relatedness, we used its disease centric projection for hierarchical correlation clustering. For data normalization, we calculated the Pearson correlation coefficient (PCC) for all pairs of diseases based on their genetic agonistic overlap with all other diseases. The clustering returned 15 disease clusters (Figure 3) and six diseases which show no or only weak correlation with any other disease. With the exception of the heterogeneous cluster 5 (hypertriglyceridemia, ovarian neoplasms, lymphoid leukemia, atopic dermatitis), the clusters mostly contain related diseases. However, many clusters also contain traits unrelated to the other phenotypes like schizophrenia in the autoimmune cluster 2. This indicates that clinical disease classifications appear to be reflected on the genetic level in general. Notably, several small clusters contain diseases which are either linked through common environmental risk factors – like smoking for lung neoplasms, peripheral vascular diseases, and chronic obstructive pulmonary disease – or present high frequencies of comorbidity, e.g. type 2 diabetes (T2D) and obesity. To get an insight into the overall extent of reported comorbidities of the diseases within the 15 clusters, we used publicly available resources [28,29] and literature mining. The within-cluster fraction of disease co-occurrence ranged from 75% to 100% (*μ* = 95.89%, *σ* = 8.66%) which provides empirical evidence of the interrelation of diseases clustered together by genetic information. Such clusters containing diseases that present high ratios of comorbidity may be potential artifacts due to “contaminated” disease cohorts including a substantial number of comorbid cases. The unbiased search for the relation of a trait marker to a disease phenotype as performed in GWAS does not distinguish between markers for a primary or related secondary (comorbid) disease. Our results suggest that this aspect may have been underestimated in some studies, albeit the presence of independently shared etiological mechanisms can naturally not be ruled out in general.

Overall, the outcome of the clustering poses the question of the extent of the influence of phenotype classification and population stratification on GWAS results. Frequent comorbidities (also of seemingly unrelated diseases such as obesity and cancer [30]), diagnostic difficulties in highly related diseases like Crohn’s disease (CD) and ulcerative colitis (UC) [31], and structural (genetic) differences in population subgroups are known to complicate GWAS and impact their outcomes [32]. With growing sample sizes in case–control studies, the potential of false positives produced by such phenomena also increases. As a response, manifold control procedures to handle these and other confounding factors have been developed which are widely used and well appreciated [32]. The heterogeneity of the clusters we retrieved once more highlights the need for the development and application of such methods.

### Odds ratio as potential indicator of primary effects

In the context of agonistic association overlap between related diseases, we used the odds ratios (ORs) reported with the SNPs to investigate their impact on the respective traits. In general, the highest ORs are reported for associations of autoimmune diseases to the HLA locus on chromosome 6. Associations with traits where few gene variants with strong effects are reported, e.g. rs6107516 in the prion protein *PRNP* associated with Creutzfeldt-Jakob disease (OR = 38.5) or rs2071348 in the hemoglobin gene cluster at 11p15.4 associated with beta-thalassemia (OR = 4.33), are exceptions from the majority of associations displaying small ORs [33,34]. Based on the effect size of variants associated with more than one trait and the same risk allele, we identified three patterns.

First, we identified variants which are likely to present general agonistic risk factors for a group of related diseases or syndromes such as rs13015714 in an interleukine receptor gene cluster at chromosome 2q12.1. This SNP is associated with celiac disease (CeD) and Crohn’s disease (CD) with equal ORs (OR = 1.19) [24,35]. In cases of frequent comorbidities, though, comparable ORs have limited informative value. The SNP rs9939609 in the *FTO* gene for instance is associated with T2D and obesity with nearly equal ORs (OR = 1.34 and OR = 1.32) and thus appears to link two coequal traits of the metabolic syndrome (we refer to the definition of the International Diabetes Foundation, 2006) [36,37]. However, for SNPs in the *FTO* gene it has been shown that adjustment for body mass index results in the loss of significance (OR∼ 1.0) of the association with T2D [38].

Second, SNPs appeared in several cases to be primarily associated with one disease, which in turn represents a risk factor for another associated trait. For instance, rs2200733 on chromosome 4q25 is linked to atrial fibrillation with a higher OR (OR = 1.72) than to stroke (OR = 1.26) [39,40]. Another example is rs964184 which is located proximal to the apolipoprotein gene cluster on chromosome 11q23 which is associated with hypertriglyceridemia with a markedly higher OR (OR = 3.28) than to coronary disease (OR = 1.13) [41,42]. The lower effects of the markers on the hypothesized “secondary sequels” may be explained by the fact that these are caused by the primary diseases, but with less than 100% penetrance.

Third, we speculate that the OR might allow conclusions with respect to the evaluation of an association in cases where similar traits are linked to the same SNP with diverging effect sizes. For instance, CD and ulcerative colitis (UC) share multiple risk loci. The two diseases are strongly related in their etiology and pathology. Thus, a clinical distinction of both diseases is difficult if based only on few criteria and might lead to inaccurate case ascertainment leading to mixed associations [31]. However, for several SNPs such as rs11209026, which is located in the *IL23R* gene, we found notably higher effects on CD (OR = 3.84) than on UC (OR = 1.74) [25,43]. Conversely, rs3024505 which lies proximal to the *IL10* gene shows a greater effect on UC (OR = 1.46) than on CD (OR = 1.12) [24,44]. Interestingly, it has been shown that *IL23* is selectively upregulated in CD while levels in UC patients are normal and *IL10* expression appears to be higher in UC as compared to CD [24,45]. Thus, the OR might – similar to the above examples – allow for identifying potentially misleading associations in closely related diseases which may result from diagnostic errors.

### Identification of branching etiologies

We searched for evidence that antagonistic signals represent genetic indicators of branching points in the etiologies of two diseases or disease groups. For the assessment of potentially multifunctional variants we therefore focused on markers with inverse effects. We identified 44 such variants, which represent almost 4% of the original association data analyzed and about 25% of the SNPs associated with more than one disease. Of those 44 variants, about one fifth (n = 9) are located in the HLA region. SNP-markers in that region are known to differ in their ability to capture the classical HLA-alleles [46] and therefore were not considered further for the present analysis.

For cases where the function of the harboring genes is known, we were able to identify conclusive models. For instance, rs2736100 in the telomerase reverse transcriptase (*TERT*) gene was reported to exert antagonistic effects in idiopatic pulmonary fibrosis (IPF) and testicular germ cell tumor (TGCT) and two other cancer traits [47-53]. Whereas telomerase activity is generally upregulated in tumors sustaining proliferation and potentiating mutagenesis and transformation of cancer cells [54], in IPF limited cell division due to decreased telomerase activity is thought to contribute to the phenomenon of high percentages of apoptotic cells in fibroblasts [55]. Consistent with that observation, disturbed telomerase activity in TGCT is believed to form a distinct mechanism of cancerogenesis in this tumor type [53]. This distinction from other cancer traits is believed to be based on the fact that testicular germ cells are the only adult cell type with high telomerase expression [56]. Another example is the telomerase RNA component *TERC*, which is essential for *TERT* functioning. Opposite alleles of SNP rs10936599 are associated with CeD and colorectal cancer (CRC) [35,57]. Jones et al. showed that rs2293607, a variant tagged by rs10936599, alone is sufficient to modulate *TERC* expression [58]. While in CRC this leads to *TERC* overexpression and longer telomeres, the opposite might apply to CeD, which exhibits telomere reduction and genomic instability [58,59]. The observation that both constituents of the telomerase complex contain independent antagonistic variants is an intriguing finding. It suggests parallel, autonomous evolution of two functionally interacting loci gone to fixation at a trade-off between early cell senescence or increased apoptosis rates (as in IPF and CeD) and oncogenesis.

A further example is rs1393350 in the tyrosinase (*TYR*) gene where the opposite alleles are linked to vitiligo and melanoma [60,61], potentially mirroring the inverse correlation observed for the two traits. The phenomenon is based on the presentation of *TYR* (self-) antigens on the cell surface of melanocytes. It is hypothesized that in vitiligo the immune system is hypersensitive towards *TYR* antigens, which are overexpressed in melanoma cells [62]. A possible explanation may be that opposite alleles differentially influence the antigenicity of the *TYR* protein, thereby conferring protection from melanoma but susceptibility to vitiligo through immune surveillance and vice versa.

In cases of functionally less or uncharacterized genes and their involvement in the associated diseases, our approach can still be used to suggest potential trait-specific effects. Antagonistic effects of rs12720356 (localized in the *TYK2* gene) in CD and psoriasis, for instance, might point towards different patterns of cytokine signaling in these two diseases [24,63,64]. Likewise, rs12727642 and rs35675666, both located in the *PARK7* gene and inversely associated with CeD and UC, could indicate differential effects of oxidative stress on each trait [25,35].

### Variant-based analysis of joint and disjoint genetic features

In this study, we identified overlapping genetic associations and their corresponding loci with analogous or contrasting effects on different diseases. We addressed the methodological challenges of the identification of the functional entities affected by GWAS-detected variants.

Associations formally implicate genomic regions which are captured via tagging SNPs representing haplotype blocks. By using the population-specific LD-based haplotype data provided by the HapMap project [65,66] or, more recently, the 1000 genomes project [67], SNP arrays are constructed aiming at a high coverage of the total genome variation, but without considering biologically functional aspects. The advantage of GWAS as a method is its unbiased approach to identify genomic regions compromised in a disease; a major drawback is that the association of markers without knowledge of the causal variants and their effects does not allow for a straightforward biological interpretation.

As we show, the reliability of an automated assignment of LD-based loci to the trait-associated variants is strongly context-dependent. Especially in cases of high gene density or, conversely, in intergenic regions/gene deserts, resolving GWAS signals is not possible without further knowledge. Simplifications such as more basic locus assignment approaches which neglect the LD structure of the genome (e.g. classifying a SNP as affecting only the most proximal gene) may seem more intuitive, might facilitate analyses and could be useful to identify causal disease-gene associations. These correct associations of genes which are detected through significant enrichment of a harbored tagging variant in a patient cohort may not be discovered when incorporating LD data in cases where the LD block of the respective variant spans across several genes. However, such approaches disregard a basic principle defining the current GWAS paradigm, namely the use of LD information in the design of genotyping arrays to achieve the genome-wide coverage of common SNPs. Hence, it can be problematic to project the variant-based GWAS data on genes or loci. Accordingly, we decided to use variant-based methods and concentrated on strong gene candidates identified via the gene function of single-gene loci whenever suggesting potential biological effects of the considered variants.

In the analysis of genetic overlaps we followed the hypothesis that the effects of variants shared across several diseases correspond to the reported risk alleles. If the risk allele is the same in all associated diseases, we assume the effect to be the same, i.e. that there is a common underlying etiology. For closely related diseases a positive correlation is not surprising, e.g. a GWAS on psoriatic arthritis (PSA) will also detect agonistic variants such as rs33980500 that are also associated with psoriasis (PS) [68,69]. Indeed, the vast majority of agonistic variants in our data set links groups of related diseases and thus may mark interesting target regions for therapeutic interventions. However, we also found a few agonistic signals connecting apparently unrelated diseases, e.g. rs6010620 which exerts susceptibility for both glioma and atopic dermatitis (AD) [50,51,70,71]. If our hypothesis is correct, an endophenotype influencing both diseases may be present which has yet to be identified. For antagonistic SNPs, on the other hand, we describe plausible mechanisms that may render variants protective against one trait and predisposing to another, labeling the affected genes/loci as pleiotropic. If pleiotropic effects are as frequent as evolutionary modelers postulate [2,72] and this effects can be identified by analyses based on GWAS, this might have great implications for the development and use of therapeutics because it would enable avoidance of potential side effects when targeting such loci. Already, there are more than 50 genotype/drug interactions known for which therapeutic dosing recommendations are available [73].

## Conclusions

Our results present new starting points for studying the genetics of complex diseases. The observation that more than 15% of the SNPs considered in our study are associated both agonistically and antagonistically with related as well as unrelated disorders indicates that the molecular mechanisms influencing causes and progress of human diseases are in part interrelated. Genetic overlaps between two diseases also suggest the importance of the affected entities in the specific pathogenic pathways and should be investigated further. These may be secondary, such as genes involved in inflammatory responses related to T2D as well as cancer [30,38]. The findings presented also demonstrate the need to clarify the relation of any phenotype linked to an associated marker. For directly interrelated diseases such as PS and PSA often PS patients without present arthritis or arthritis in the past are used as additional control group. Associations are then interpreted as PSA-specific if not as strongly associated with PS [74,75]. Comparable procedures may proof useful in frequently co-occurring diseases genetically linked by agonistic variants. Nevertheless, the complex genetics of multifactorial diseases asks for a better understanding of the functions underlying common disorders. An improved characterization of the endophenotype, such as metabolite or protein concentrations, may enhance our understanding of identical pathomechanisms that link agonistic genetic loci to clinically distinct traits. Pleiotropic effects, on the other hand, that are harbored in the same locus may trigger different mechanisms interfering with the genetic or environmental background. The detailed examination of antagonistically associated loci may thus lead to first insight into the mechanism of the various types of pleiotropy in human diseases.

## Methods

### Association selection and curation

**Figure 4:**
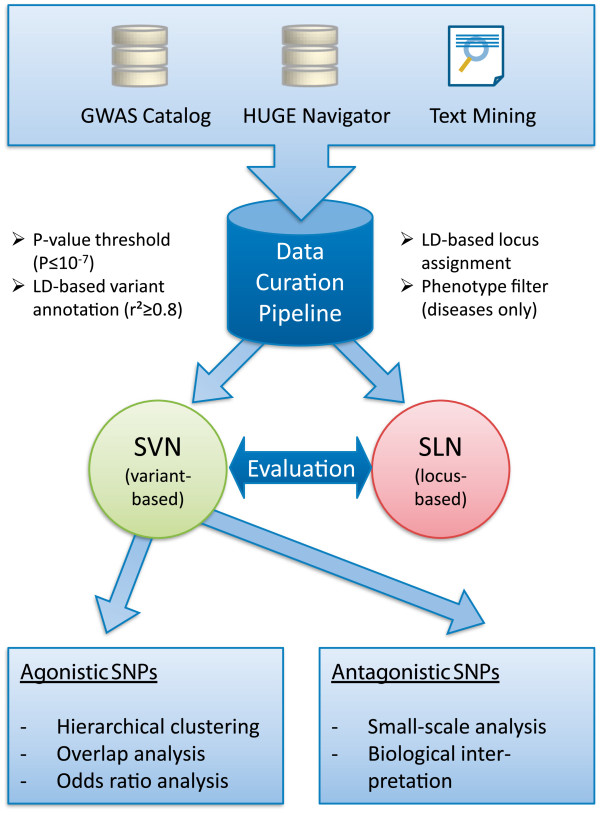
**Data prioritization and analysis workflow.** We established a semi-automated curation pipeline which automatically gathers and annotates GWA data obtained from three sources (locus assignment included). Last step of the preprocessing was the manual inspection of risk alleles and odds ratios. With this data set at hand, we construct a locus-based (SLN) and a variant-based (SVN) network representation of the data. For quality reasons, we then limited analyses to the SVN and investigated the contained variants and their effects further.

We obtained the core list of candidate sentinel SNPs from ‘A Catalog of Published Genome-Wide Association Studies’ [1] accessed on June 30, 2011 ( http://www.genome.gov/gwastudies). Additional associations where retrieved from HuGE Navigator [76] and automated Text Mining [77]. New (i.e. not contained in the GWAS Catalog) association markers were manually tested on compliance with the criteria for inclusion in the GWAS Catalog before insertion in the candidate list. Associations with copy number variants (CNVs) as well as with pending SNPs were removed. For consistency, SNP identifiers were mapped to the RefSNP numbers of the same dbSNP release (build 131, http://www.ncbi.nlm.nih.gov/snp). For the same reason we semi-automatically translated trait descriptions to the official terms given in the Medical Subject Headings (MeSH). In this process, associations with quantitative and non-disease traits were eliminated. Finally, we lowered the association P-value threshold from 10^−5^ (as in the GWAS Catalog criteria) to 10^−7^ as to reduce the potential of artificial associations. The workflow of the methods of our approach is sketched in Figure 4.

### Construction of GWAS networks

For the construction of the locus-based data representation, we defined an associated locus as the whole genomic region captured by SNPs in strong LD, *r*^2^ ≥ 0.8, with the marker originally reported in a GWAS contained in our data set. The locus is then characterized as all genes located within this genomic region (referred to as “gene locus”) (Figure 2). If the region contains no genes, the locus is assigned to its chromosomal location (referred to as “intergenic locus”). LD data and gene information were obtained with the SNAP tool [78]. After locus assignment, our final data set consisted of 111 different traits linked via 1,120 SNPs to 508 gene loci and 226 intergenic loci.

Based on this list we constructed a bipartite graph consisting of two disjoint sets of nodes (Figure 1A) representing the complete association data. The first node set corresponds to the traits, whereas the other set comprises the associated loci. Two nodes are connected by an edge if a variant within the respective locus is associated with the corresponding trait. By removal of isolated traits, i.e. traits which share no associated locus with another trait (*n* = 27) (Figure 1A), and cutting out loci which are associated with only one trait (*n* = 577), we retrieved the SLN (Figure 1B).

To obtain a variant-based representation of the data, we repeated the network generation on marker scale by utilizing the set of variants associated with more than one distinct trait. For this, we used the LD data to mutually assign the associated traits of sentinel SNPs in pairwise LD if not already present. In other words, each variant is, in addition to its own associated traits, assigned the traits associated with all correlated SNPs. This set consists of 175 SNPs located in 94 loci and associated with 55 diseases (Additional file 2: Table S1). In the resulting bipartite SVN, a trait and a locus are linked if the locus contains a variant which comprises associations with this and at least one other trait. Here, the allele information was included in the graph visualization by coloring of the edges (Figure 1C). Both the SLN and the SVN are provided as machine-readable files, see Additional files 5 and 6.

### Network analysis

The network concepts which we used to compare the properties of the SLN and the SVN are defined as given in [79]:

(1) $$\;Density=\frac{\sum_{i}\sum_{j\neq i}a_{\mathrm{ij}}}{n\left(n-1\right)}=\frac{mean\left(k\right)}{n-1}$$

where *a*_*ij*_ = 1 if nodes *i* and *j* are connected and 0 otherwise. *mean*(*k*) denotes the mean connectivity, which for a node *i* is defined as $k_{i}=\underset{j\neq i}{\sum }a_{\mathrm{ij}}$.

(2) $$\;Centralization=\frac{n}{n-2}\left(\frac{\max\left(k\right)}{n-1}-Density\right)$$

(3) $$\;Heterogeneity=\frac{\sqrt{variance\left(k\right)}}{mean\left(k\right)}$$

To automatically distinguish the two node sets contained in the SVN, we used directed edges. Direction is always from disease (source) to locus (target). The distinct node degree distributions thus are identical to the indegree distribution and the outdegree distribution. The topological coefficient as a measure of modularity [80]*T*_*i*_ of a node *i* is defined as:

(4) $$\;T_{i}=\{\begin{array}{c} 0,\;if\;N_{i}<2\;\\ \text{avg}\left(\frac{S\left(i,j\right)}{N_{i}}\right),\;else \end{array}$$

where *N*_*i*_ is the number of neighbors of *i* and *S*(*i*, *j*) is the number of shared neighbors of nodes *i* and *j* (undefined if *i* and *j* do not share a neighbor) plus one if *j* is a neighbor of *i*.

Power-law functions of the form *y* = *e*^*a*^*x*^*b*^ were fitted using least squares fitting where the coefficients are defined as $b=\frac{n\sum_{i=1}^{n}\left(\ln x_{i}\ln y_{i}\right)-\sum_{i=1}^{n}\left(\ln x_{i}\right)\sum_{i=1}^{n}\left(\ln y_{i}\right)}{n\sum_{i}^{n}{\left(\ln x_{i}\right)}^{2}-{\left(\sum_{i=1}^{n}\ln x_{i}\right)}^{2}}$ and $a=\frac{\sum_{i=1}^{n}\left(\ln y_{i}\right)-b\sum_{i=1}^{n}\left(\ln x_{i}\right)}{n}$. As goodness-of-fit measure, we give the coefficient of determination

(5) $$\;R^{2}={\left(\frac{n\sum_{i=1}^{n}x_{i}y_{i}-\sum_{i=1}^{n}x_{i}\sum_{i=1}^{n}y_{i}}{\sqrt{n\sum_{i=1}^{n}{x_{i}}^{2}-{\left(\sum_{i=1}^{n}x_{i}\right)}^{2}}\sqrt{n\sum_{i=1}^{n}{y_{i}}^{2}-{\left(\sum_{i=1}^{n}y_{i}\right)}^{2}}}\right)}^{2}$$

for the linear transformation of the power-law functions, i.e. ln *y* = *a* + *b* ln *x*.

### Determination of agonistic and antagonistic effects

For all variants associated with more than one trait, we manually extracted the risk alleles (OR > 1, independently of major or minor allele status) and odds ratios from the reporting studies. The alleles were mapped to the forward DNA strand according to dbSNP 131. The same procedure was applied to markers which were indirectly associated with a trait over LD. If for all traits the same associated risk allele (and corresponding allele, respectively) was reported, the SNP was classified as agonistic. If the risk alleles of a SNP were opposed in the associated diseases, the variant was classified as antagonistic.

### Genetic clustering

We applied complete-linkage hierarchical clustering to identify groups of traits genetically overlapping with respect to agonistic signals. Normalization was performed using the linear PCC defined as $\rho_{X,Y}=\frac{\mathrm{cov}\left(X,Y\right)}{\sigma_{X}\sigma_{Y}}$ where the input are the vectors of the variant-based agonistic overlap of two distinct diseases *X* and *Y* to all other diseases. Thus, disorders which are clustered together show a homogeneous association overlap pattern to all other diseases, while diseases which are not clearly assigned to a cluster present a more heterogeneous pattern relatively unique in the SNP data. For cluster definition, we used a Euclidian distance threshold of 1.71. This threshold was determined as the maximal distance at which the six traits not correlating with other diseases (Figure 3) remain non-clustered.

### Calculation of the CPMA statistic for autoimmune loci

We downloaded the dataset S1 from [8] and extracted the information on autoimmune-linked SNPs contained in the SVN. We used the Z-scores given in the file to compute two-sided P-values for all seven GWAS. Using the CPMA code provided on http://www.cotsapaslab.info/index.php/software/cpma/ we calculated the CPMA P-values as described in [8].

## Competing interests

The authors have nothing to declare.

## Authors’ contributions

Conceived and designed the study, curated and analyzed the data, wrote the manuscript: MA, MLH, HB, SW. Curated the data, wrote and critically revised the manuscript: ER. Delivered and interpreted data: BW. Contributed to manuscript writing: AF, MK, AP, HWM. Interpreted the data: JW. Curated and interpreted data: MR. Conceived the study: TI, VS. All authors read and approved the final manuscript.

## Supplementary Material

Additional file 1 **Figure S1.** Disease-centric projection of the SVN. The SVN (see Figure 1C) is transformed in a network consisting of diseases only. Here, two traits are connected if they are associated with the same variant. The colors of the disease nodes correspond to disease classes according to the MeSH ontology, multi-colored nodes indicate an association with different disease classes. The node size reflects the number of traits a disease has shared associations with. The direction of the shared variants is indicated by the edge color reflecting the corresponding allelic information: gray indicates agonistic variant(s), red corresponds to antagonistic variant(s), and blue mark both agonistic and antagonistic signals in the two corresponding traits. Click here for file

Additional file 2 **Table S1.** List of all disease-variant associations contained in the SVN. Contained is the high-quality data set which was used for the construction of the SVN, ordered by the rs-number of the tagging SNP. The first column contains this rs-number of the tagging SNP, the second column lists the disease associations and the third column gives the PubMed ID of the GWAS publication the association was reported in. In the fourth column the (gene or intergenic) locus of the tagging SNP can be found. The sixth column gives the SNP and the risk allele reported in the GWAS. If the rs-numbers of the tagging SNP (column 1) diverges from the rs-number listed here, the association was assigned via LD. For these cases, in column seven the corresponding allele of the tagging SNP is given, followed by the P-value and the odds ratio reported with the SNP (i.e. the reported SNP in column six). Blue row-coloring identifies non-HLA located antagonistic SNPs, while rows containing agonistic SNPs are not colored. Rows in green list antagonistic SNPs in the HLA region (not considered in the manuscript). Tagging SNPs which we included in our rationale are marked in bold red font. Click here for file

Additional file 3 **Table S2.** CPMA P-values for autoimmune-linked SNPs and their corresponding loci in the SVN. Listed are all SNPs contained in Supplementary Table 1 for which association data could be obtained from [8]. The second column gives the LD-based loci of the SNPs as used in the SVN. The third column contains the CPMA P-Values. Click here for file

Additional file 4 **Figure S2.** Network properties of the SVN. A: The log-log-plot of the degree distribution of the SVN follows a power-law (*γ* = 1.32; R^2^ = 0.69) and therefore attributes the SVN to be scale-free and, thus, non-random. B: The modular structure of the SVN was confirmed by the topological coefficient which follows a power-law distribution on a log-log-scale. When considering the two node types separately, in both cases a scale-free topology can be identified: C: disease nodes (*γ* = 0.97; *R*^2^ = 0.71) and D: locus nodes (*γ* = 2.98; *R*^2^ = 0.93). Click here for file

Additional file 5 **Graph data of the SLN in yEd graphml format. **View with yEd ( http://www.yworks.com/en/products_yed_about.html). Using yEd, the file can be converted to GML format which is readable by Cytoscape ( http://www.cytoscape.org/). Click here for file

Additional file 6 **Graph data of the SVN in yEd graphml format.** View with yEd ( http://www.yworks.com/en/products_yed_about.html). Using yEd, the file can be converted to GML format which is readable by Cytoscape ( http://www.cytoscape.org/). Click here for file
