# Supplementary figures and images for: Network-based SNP meta-analysis identifies joint and disjoint genetic features across common human diseases
Source: BMC Genomics. 2012 Sep 18;13:490. doi: 10.1186/1471-2164-13-490 (PMC3782362; doi:10.1186/1471-2164-13-490)

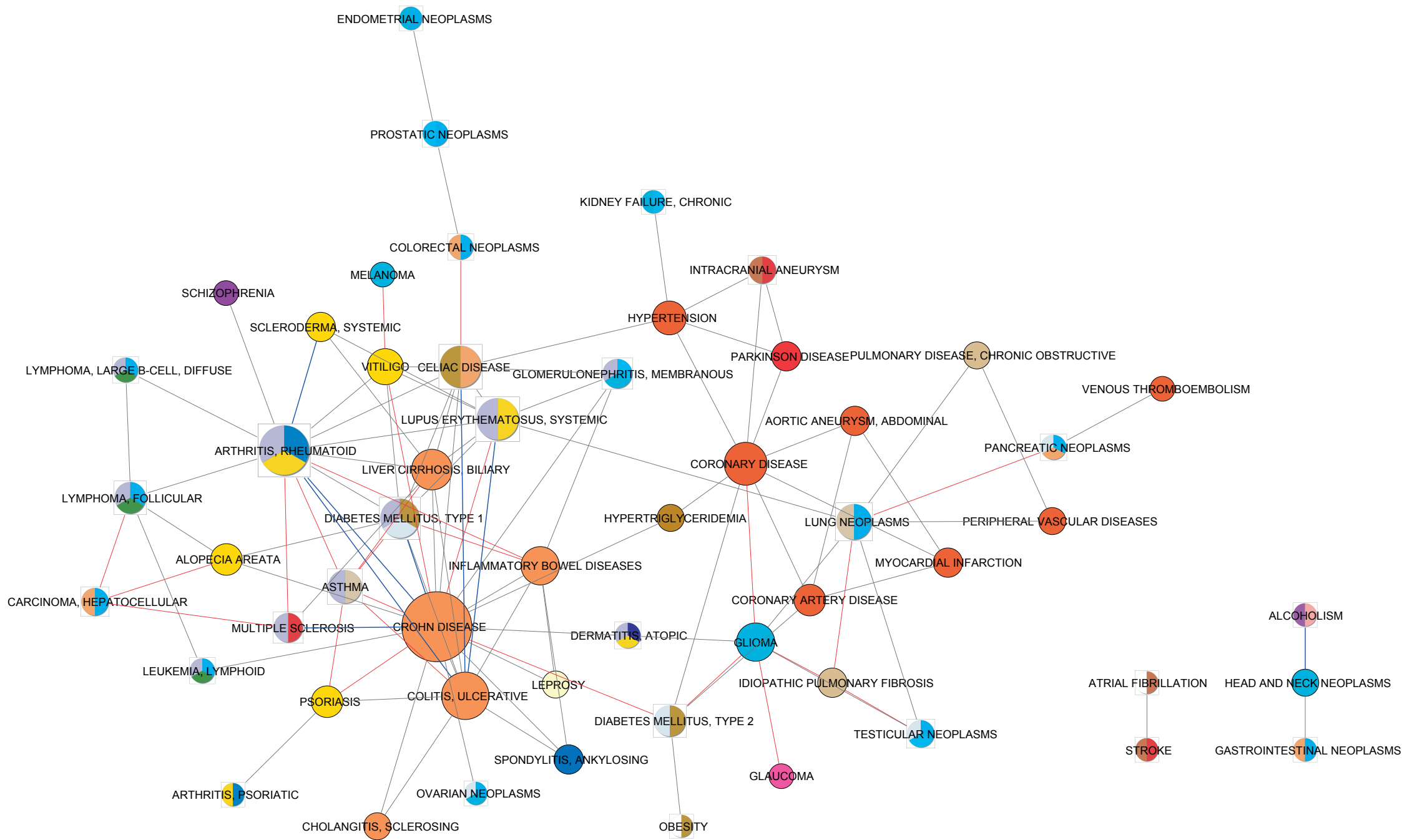

Supplement: Additional file 1 — Figure S1. Disease-centric projection of the SVN. The SVN (see Figure 1C) is transformed in a network consisting of diseases only. Here, two traits are connected if they are associated with the same variant. The colors of the disease nodes correspond to disease classes according to the MeSH ontology, multi-colored nodes indicate an association with different disease classes. The node size reflects the number of traits a disease has shared associations with. The direction of the shared variants is indicated by the edge color reflecting the corresponding allelic information: gray indicates agonistic variant(s), red corresponds to antagonistic variant(s), and blue mark both agonistic and antagonistic signals in the two corresponding traits. [file 1471-2164-13-490-S1.pdf]

A

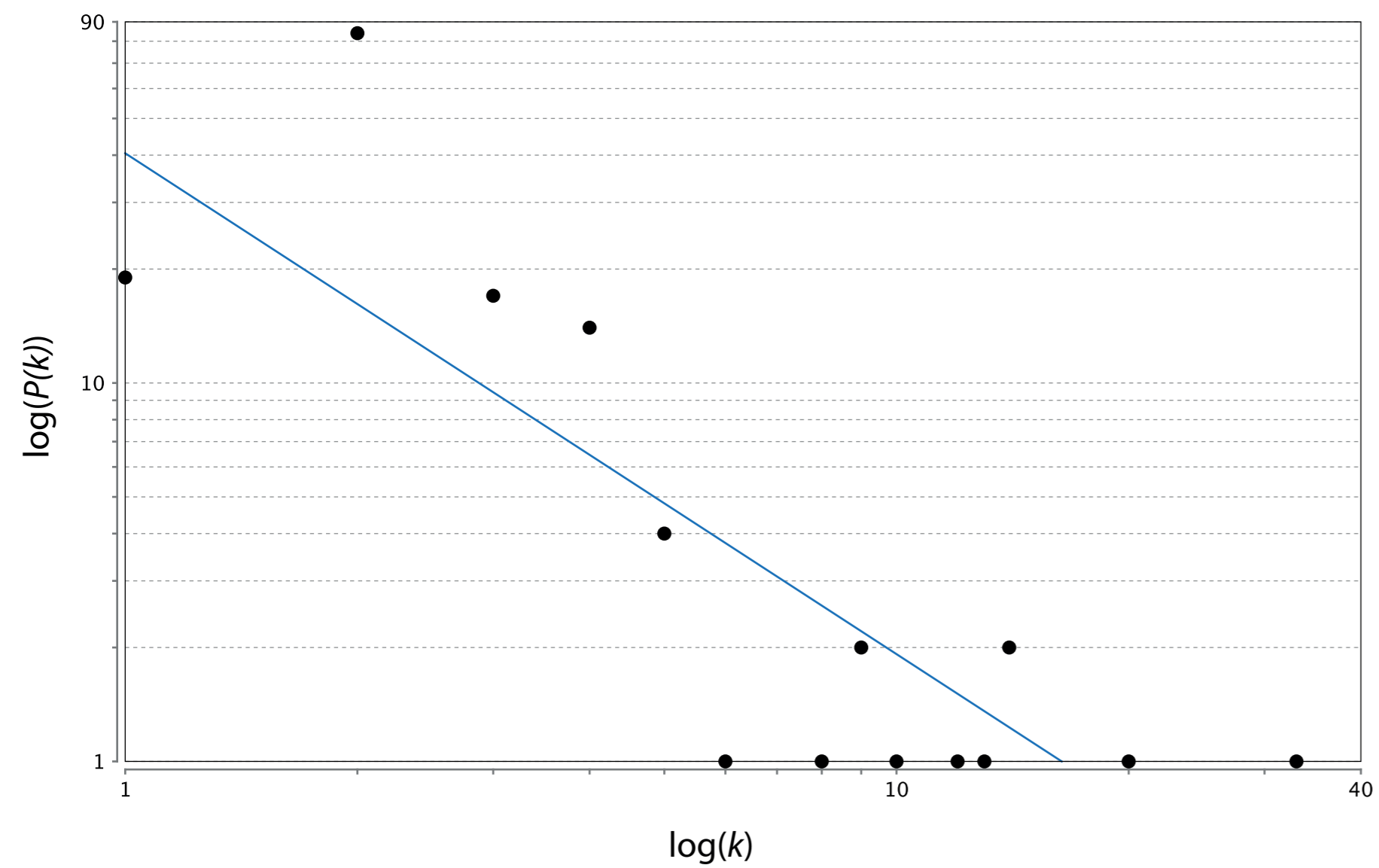

B

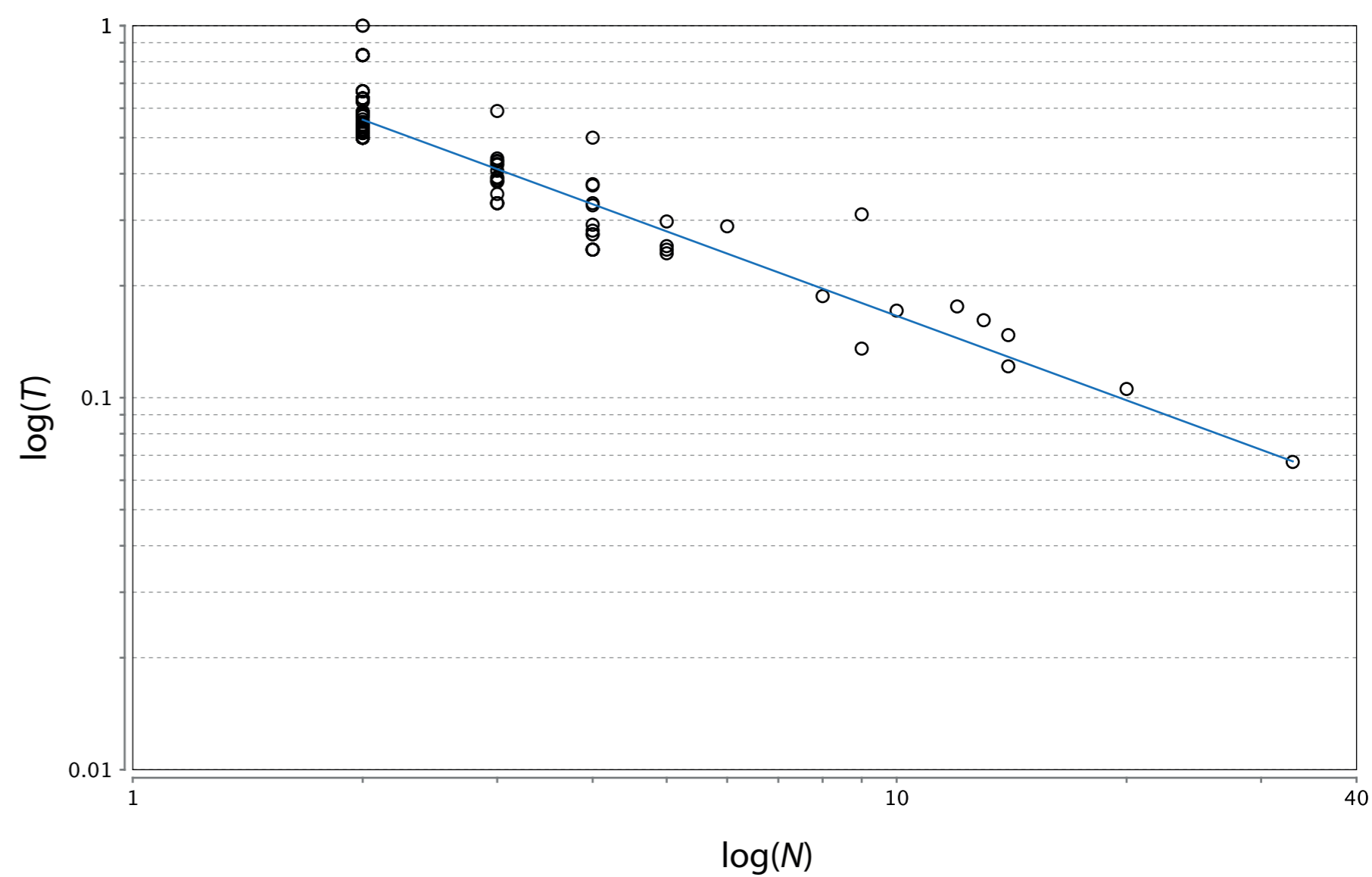

C

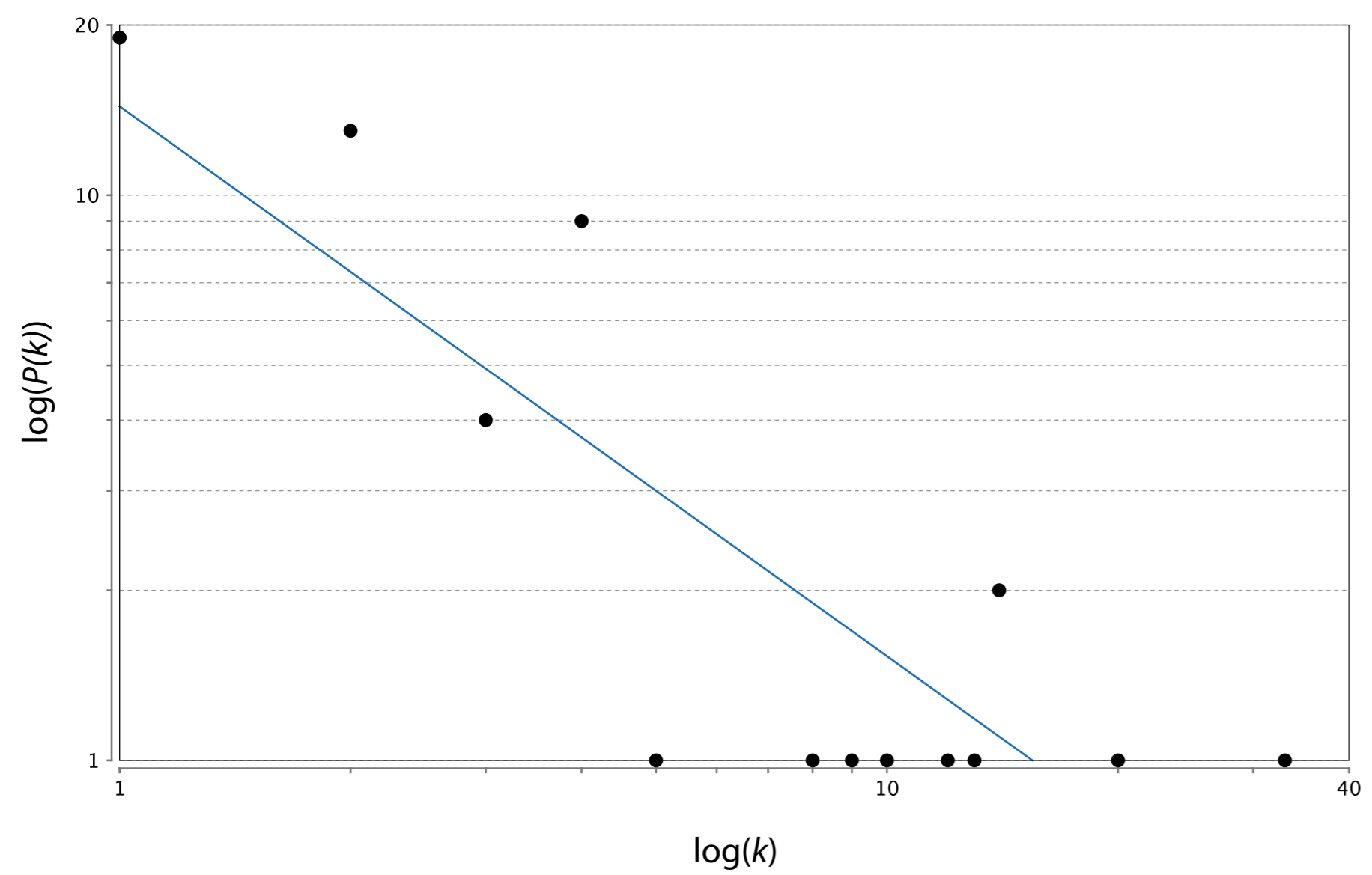

D

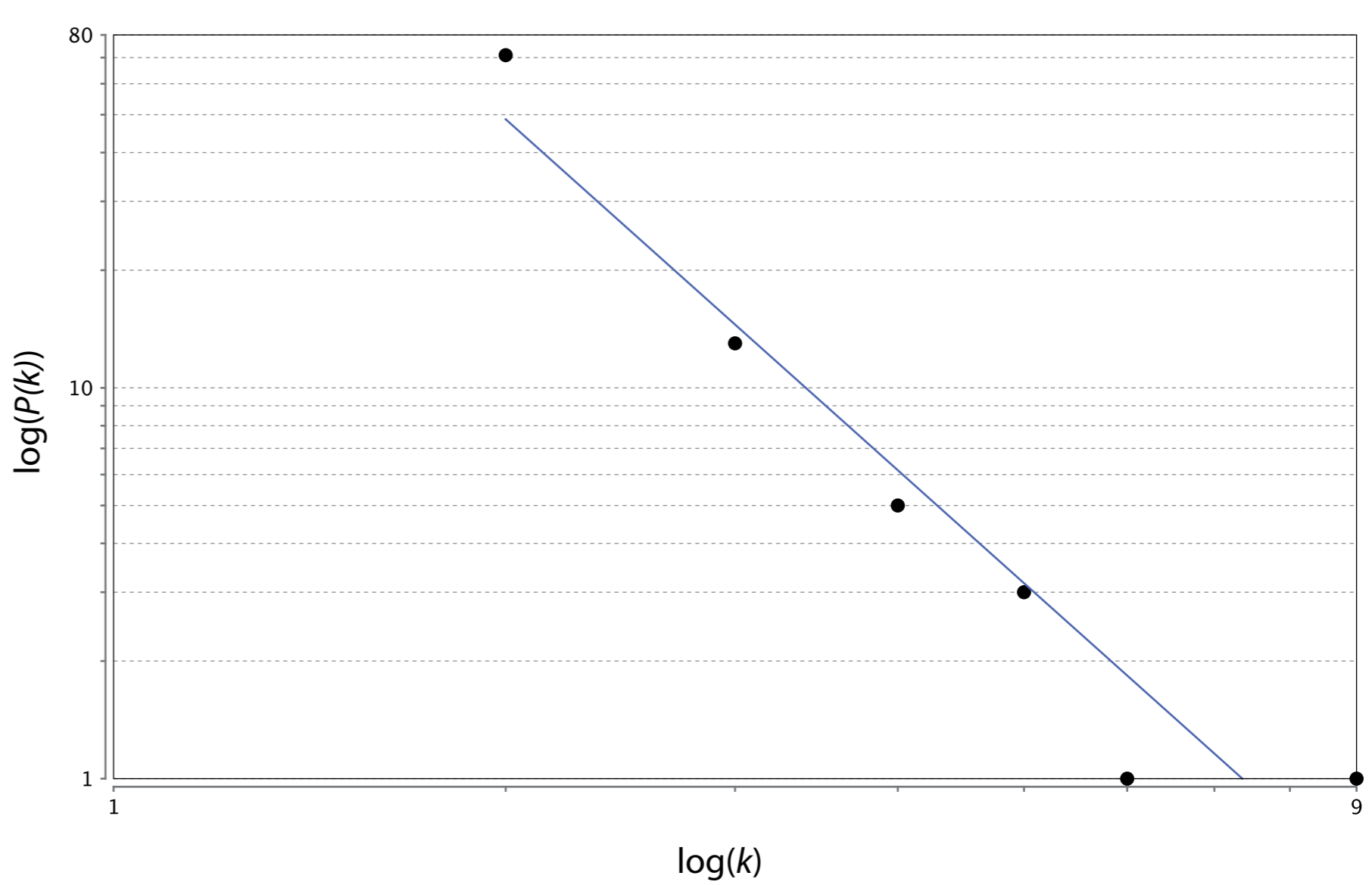

Supplement: Additional file 4 — Figure S2. Network properties of the SVN. A: The log-log-plot of the degree distribution of the SVN follows a power-law (γ = 1.32; R2 = 0.69) and therefore attributes the SVN to be scale-free and, thus, non-random. B: The modular structure of the SVN was confirmed by the topological coefficient which follows a power-law distribution on a log-log-scale. When considering the two node types separately, in both cases a scale-free topology can be identified: C: disease nodes (γ = 0.97; R2 = 0.71) and D: locus nodes (γ = 2.98; R2 = 0.93). [file 1471-2164-13-490-S4.pdf]
